# Brain-to-BAT - and Back?: Crosstalk between the Central Nervous System and Thermogenic Adipose Tissue in Development and Therapy of Obesity

**DOI:** 10.3390/brainsci12121646

**Published:** 2022-12-01

**Authors:** Andreas Till, Charlotte Fries, Wiebke K. Fenske

**Affiliations:** Department of Internal Medicine I, Division of Endocrinology, Diabetes and Metabolism, University Medical Center Bonn, D-53127 Bonn, Germany

**Keywords:** obesity, adipocytes, UCP1, SNS, hypothalamus, bariatric surgery, RYGB, leptin, ghrelin, insulin, glucagon, GLP-1, GIP, secretin, batokines, diabetes, gut microbiome

## Abstract

The body of mammals harbors two distinct types of adipose tissue: while cells within the white adipose tissue (WAT) store surplus energy as lipids, brown adipose tissue (BAT) is nowadays recognized as the main tissue for transforming chemical energy into heat. This process, referred to as ‘non-shivering thermogenesis’, is facilitated by the uncoupling of the electron transport across mitochondrial membranes from ATP production. BAT-dependent thermogenesis acts as a safeguarding mechanism under reduced ambient temperature but also plays a critical role in metabolic and energy homeostasis in health and disease. In this review, we summarize the evolutionary structure, function and regulation of the BAT organ under neuronal and hormonal control and discuss its mutual interaction with the central nervous system. We conclude by conceptualizing how better understanding the multifaceted communicative links between the brain and BAT opens avenues for novel therapeutic approaches to treat obesity and related metabolic disorders.

## 1. Brown Adipose Tissue (BAT) Architecture and Thermogenic Function

### 1.1. Introduction: Metabolism Matters!

The year 2016 represents a dramatic and alarming turning point in the history of humankind: According to the World Health Organisation (WHO), for the first time there are more people killed by overweight than by underweight. With an incredible 2 billion adults being overweight and 650 million obese (as defined by Body Mass Index/BMI ≥ 30 kg/m^2^), the world is likely to harbor more than one billion obese adults by 2025, a health burden which is a dramatic menace for the world-wide health systems. While this alarming trend is global (with the exception of sub-Saharan Africa and Asia), obesity itself and the entirety of associated comorbidities is, of course, preventable. Understanding how the human body orchestrates the input of dietary fuels and output of energy and thus facilitates homeostasis (and why this obviously does not work efficiently in obese patients) will be key to face the upcoming obesity pandemic and to design smart prevention and treatment strategies. Luckily, groundbreaking basic and translational research in the area of metabolism and metabolic diseases has sparked the scientific interest in one peculiar (and often overlooked) tissue which eventually could turn into the most attractive target to fight the imminent health crisis. This review article focusses on the function and complex regulation of this promising and exciting metabolic organ.

### 1.2. Overview of BAT Discovery and Architecture

Homoeothermic animals require tight control of their core body temperature in order to keep up organismal functions and avoid systemic damage by hypothermia. With the intention to investigate the cellular basis for this vital function, a peculiar tissue consisting of fat cells (adipocytes) with numerous small lipid droplets and an unusually high number of mitochondria was discovered in the interscapular region at the back of marmots. Due to the high iron content of the mitochondria and the distribution of lipid droplets throughout the cytoplasm, this tissue appears histologically dark red to brown why it was referred to as ‘Brown Adipose Tissue’ (BAT). This designation contrasts with the White Adipose Tissue (WAT, located at the abdomen, around the waist and thighs), which usually contains only limited mitochondrial mass and one big liposome conveying its typical yellowish appearance. While WAT is well recognized as the main tissue for storing excess calories in the form of lipids (i.e., triglycerides/TG), BAT was long thought to be exclusively devoted to the regulation of body temperature during hibernation. It took until the beginning of the 20th century to discover that the BAT is not only a heat-producing organ in hibernating mammals but is also regulating thermogenic function in non-hibernating organisms in response to external stress conditions, i.e., acute cold stress and long-term cold acclimation (for review see [1]). How far BAT thermogenic activity also contributes to hyperthermia during fever is still a subject of ongoing debate (see below).

**Figure 1:**
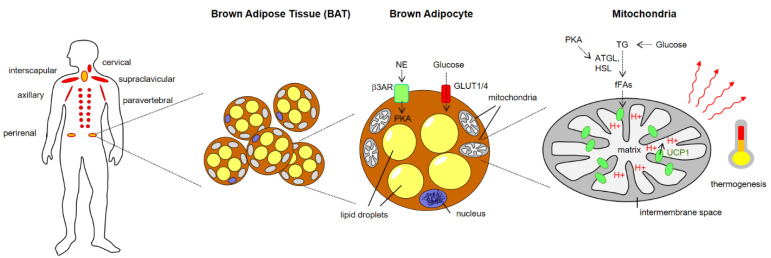
Brown Adipose Tissue (BAT) localization, architecture and role in thermogenesis. ATGL, adipose triglyceride lipase; fFAs, free fatty acids; GLUT, Glucose transporter; HSL, hormone-sensitive lipase; NE norepinephrine, PKA, protein kinase A, TG, triglycerides.

Technical advances in the field of imaging technology, particularly hybrid positron emission tomography/computed tomography (PET-CT), and functional analyses using traceable glucose analogs (such as 18F-fluoro-2-deoxy-d-glucose/FDG), have greatly contributed to unraveling more important details on BAT morphology and function. The relative mass and distribution of BAT dramatically change during early development and across the entire life span. Neonatal mammals and infants are largely protected against cold stress due to dense BAT depots within their interscapular regions. In contrast, adult mammals (in addition to hibernating species) were long time believed to harbor only minor remnants of BAT without physiological relevance. In this regard, the years 2007–2009 represent a major turning point since several groups independently demonstrated the presence, functional activity and metabolic plasticity (i.e., the ability to adjust metabolically to external conditions) of BAT conserved in adult humans [2,3,4] with six main anatomical storage sites in the human organism: cervical, supraclavicular, mediastinal, paravertebral, axiallary and abdominal [5] (Figure 1).

### 1.3. Metabolic Function of BAT

BAT is now recognized as the main tissue in the organism for transforming chemical energy (stored in the form of lipids) into thermic energy (i.e., heat). This process, referred to as ‘non-shivering thermogenesis’ (as opposed to the shivering of muscles that generates heat as a byproduct of muscle activity), fulfills at least two important functions: First, it represents a safeguarding mechanism both under short-term cold stress and long-term cold acclimation conditions to protect key organs from hypothermic damage by warming the blood flow. Second, BAT thermogenesis is a major regulatory process ensuring systemic metabolic homeostasis by increasing glucose uptake and combusting energy in response to surplus nutrient conditions (reviews: [6,7]). In addition, recent research has shown that the physiological role of BAT is not only to produce heat but also to act as an endocrine/paracrine organ, secreting molecules that affect systemic physiology and thus shape whole-body metabolism. The group of these secretory factors (collectively named **‘batokines’** [8,9]) comprises various types of signaling molecules, including peptides (such as FGF-21, IGF-1, follistatin, IL-6 and RBP-4), lipid-based metabolites (e.g., 12,13-dihydroxy-9Z-octadecenoic acid) and even exosomal microRNAs (e.g., miR-99b). Batokines enhance the thermogenic capacity by promoting BAT hypertrophy, adipose tissue vascularization and WAT beiging, but they also exert long-distance control of metabolism—particularly whole-body glucose and lipid disposal—by conveying systemic signaling cues to metabolic organs. Moreover, they mediate the general metabolic activity of the liver, heart and muscle, and affect vascularization, WAT/BAT innervation and immune functions [10,11,12].

Given this central role of BAT for both energy dissipation and endocrine/paracrine control of metabolic balance, significant attention has been directed within the last years toward exploiting BAT thermogenic function for the treatment of obesity and associated comorbidities. In fact, there are several interesting arguments for targeting BAT activity in the development of novel therapies for weight management and metabolic diseases. First, concerning the overall metabolic function of BAT, it was demonstrated that cold-induced BAT activity as determined by (18)FDG-PET correlates with human leanness [3] and that BAT-positive persons at (18)FDG-PET had lower visceral and subcutaneous abdominal adipose tissue and liver fat content than BAT-negative persons [13]. Moreover, subjects with more active BAT exhibit better metabolic profiles [14], including lower fasting glucose levels [15] and higher insulin-stimulated glucose disposal [16], as well as reduced arterial inflammation and resulting arteriosclerosis [17]. Notably, a recent largescale epidemiological study indicates that BAT presence inversely correlates with hyperlipidemia, type 2 diabetes (T2D) and major cardiometabolic diseases [18]. Overall, it was reported that a fully-activated BAT organ could dissipate energy equivalent to approximately 4 kg of WAT over one year [4,15]. Consequently, current evidence suggests that BAT-dependent energy combustion significantly contributes to whole-body energy expenditure and that organ activity is linked with multiple beneficial effects on energy homeostasis and metabolic outcome parameters. As a consequence, recruiting BAT volume and boosting its activity may represent an attractive target for the development of novel pharmacological drugs against obesity and its comorbidities such as cardiovascular disease, diabetes and cognitive decline (see Section 3).

### 1.4. Molecular Mechanisms of BAT Thermogenesis

However, how exactly do brown adipocytes accomplish their job? At the cellular level, the greatest energy transformation in humans and other eukaryotes occur in the mitochondria. Here, the electrochemical gradient of protons (H^+^) across the inner membrane of the respiratory chain usually drives the phosphorylation of ADP to ATP via the activity of the F0/F1-ATP synthase. Therefore, under physiological conditions, respiration is directly coupled to ATP production. In BAT adipocytes, this fundamental mechanism is hijacked and exploited to translate chemical energy into thermic energy by bypassing (‘uncoupling’) the electron transport from ATP production (see [19] for an excellent review). The most prominent and best-characterized effector of this process is a protein termed cold-inducible mitochondrial uncoupling protein 1 (UCP1) [19,20,21]. 

UCP1 (also known as thermogenin) represents a member of the large mitochondrial anion carrier family whose members facilitate the shuttling of ions across mitochondrial membranes. It is a multi-pass transmembrane protein spanning the inner membrane within mitochondria. In the presence of free fatty acids (derived from TG breakdown by lipolysis), UCP1-channels mediate the leakage of H^+^ ions from the intramembrane space to the mitochondrial matrix, thereby uncoupling the electron transport from ATP synthesis [20,22] (Figure 1). As a consequence of this mitochondrial short-circuit, heat is produced within mitochondria and distributed across cell and tissue barriers via the blood stream to increase the body core temperature.

The canonical activation pathway for cold- and diet-induced BAT thermogenesis relies on neurotransmitter-mediated activation of β-adrenergic receptors (mainly of the β3 subtype, β3ARs) on brown adipocytes [23]. Engagement of these G-protein coupled receptors leads to cAMP-mediated activation of protein kinase A (PKA), which activates adipocytic lipases (such as adipose triglyceride lipase/ATGL and hormone-sensitive lipase/HSL) that convert triglycerides (TG) into free fatty acids (fFAs). Synthesis of fFAs from TG in BAT is further fueled by glucose uptake and generation of TG from glucose catabolism via pyruvate and acetyl-CoA. After the import into the mitochondria, fFAs bind to UCP1 and trigger steric changes of UCP1 binding domains that affect its tridimensional conformation, eventually resulting in leakage of protons into the matrix and subsequent heat production.

Importantly, while UCP1-dependent mitochondrial uncoupling appears to be the most prominent pathway for thermogenesis in BAT (mainly induced by cold and nutrient surplus), recent reports have identified interesting alternative pathways that act either in concert or entirely independent of UCP1. Adipocytes within the WAT that acquire brown-like features upon appropriate stimulation (sometimes referred to as beige or brown-in-white/‘brite’ adipocytes) have been shown to exploit a calcium cycling mechanism selectively for heat production. This mechanism relies on Ca^2+^ ATPase 2b, an ATPase that represents the pivotal pump for sequestering calcium from the cytosol into the endoplasmatic reticulum (ER) and thus is involved in ER stress responses [24]. Moreover, it has repeatedly been demonstrated in both beige and brown adipocytes that the mitochondrial Creatine Kinase and its substrate Creatine elicit an ATP → ADP turnover cycle that results in thermogenesis by dissipation of stored energy and that this mechanism is downstream of canonical β3-adrenergic signaling, thus acting in parallel to UCP1 (for review, see [25]). Finally, a UCP1-independent but fatty acid-activated pathway has been described by Bertholet and colleagues that utilizes the ADP/ATP carrier (AAC) located at the mitochondrial inner membrane. Patch-clamp measurements of isolated mitochondria could demonstrate pronounced proton leaks dependent on AAC in the absence of UCP1 [26].

In general, the concept is emerging that molecular mechanisms that result in a decrease in the cellular ATP/ADP ratio convene on exerting thermogenic activity, either via UCP1 or in a UCP1-independent fashion.

In the following paragraph, we would like to review current knowledge about how the Central Nervous System (CNS), together with other signaling pathways, is equipped to regulate BAT activity in a bidirectional manner systemically, and how these regulatory pathways adapt during the process of metabolic disease, as such might offer interesting options for complementary management of obesity and related diseases.

## 2. Control of BAT Activity by the CNS, SNS and Endocrine Signals

### 2.1. Neuronal and Endocrine Control of BAT Activity

**Figure 2:**
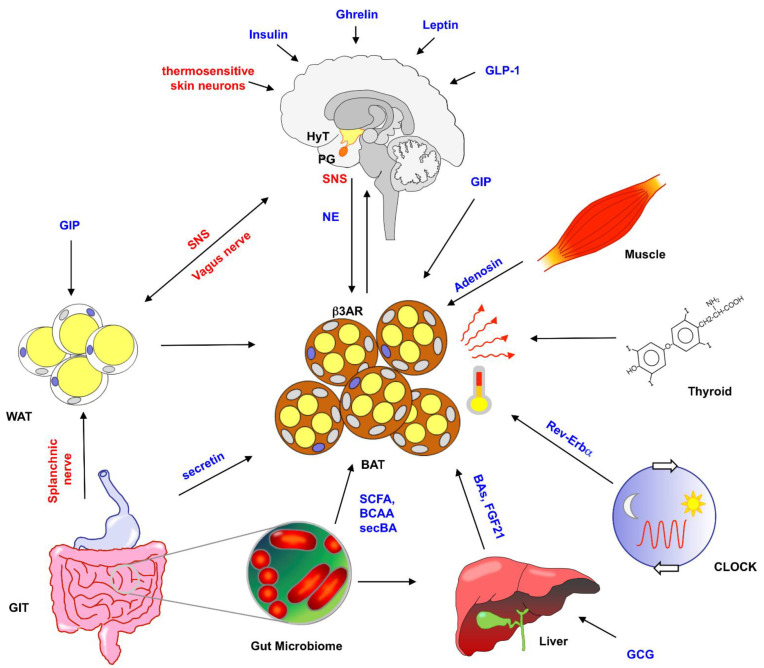
Multifaceted regulation of BAT activity. Thermogenic activity in brown adipose tissue (BAT) is controlled in response to cold exposure or caloric excess via various control mechanisms. These include the Sympathetic Nervous System (SNS) originating within the hypothalamus, crosstalk with White Adipose Tissue (WAT), modulation by thyroid hormones, transcriptional regulation by the circadian rhythm and input from liver, muscles and gastrointestinal tract (GIT). The latter mechanism can be mediated either directly via the secretion of gut hormones such as secretin by GIT cells or by alterations in the gut microbiome and subsequent changes in microbial metabolite profiles within the GIT or the circulation (with long-ranging effects on the entire body). Neuronal signaling routes are displayed in red, and molecules exerting signaling effects are shown in blue. BAs, bile acids, BCAA, branch-chained amino acids; GCG, Glucagon; GIP, gastric inhibitory peptide; GLP-1 Glucagon-like protein-1; HyT, hypothalamus; NE, norepinephrine; SCFA, short-chained fatty acids; secBA, secondary bile acids; SNS, sympathetic nervous system; PG, pituitary gland.

Given the pivotal importance of energy homeostasis for the organism, it is not surprising that BAT activity is regulated by various pathways that jointly integrate environmental as well as internal signals in order to fine-tune thermogenesis and nutritional energetics in an orchestrated manner [27,28] (Figure 2). Owing to its high oxygen consumption, BAT is a highly vascularized tissue but also contains a high density of unmyelinated nerve fibers that provide stimulation by the sympathetic nervous system (SNS). The CNS plays a key role in sensing and controlling the energy status of the organism, while the hypothalamus, in particular, has emerged as an integrating, superordinate master regulator of whole-body energy homeostasis and expenditure. SNS innervation of BAT is largely facilitated by the autonomous secretion of neurotrophic factors by the target organ, but it has recently been shown that γδ-T cells significantly contribute to BAT sympathetic innervation by stimulating expression of neurotrophic TGF-β via the key T cell cytokine IL-17 [29]. Adipose tissue thermogenesis is highly dependent on the SNS outflow derived from specific regions of the brain and the hypothalamus, whose neurons terminally secrete the postganglionic neurotransmitter norepinephrine (NE), which binds to and activates β3-adrenergic receptors (β3ARs) on brown adipocytes. As outlined above, this receptor activation essentially triggers BAT lipolysis and UCP1-dependent thermogenesis.

It is important to note that, in addition to β3AR signaling, alternative ligand/receptor systems for activation of BAT have been identified: Using animal models and both genetic and pharmacological intervention, Thorsten Gnad and colleagues from Alexander Pfeifer’s group demonstrated convincingly that engagement of adenosine receptors A2A and A2B can also efficiently activate BAT activity and differentiation (‘browning’) of WAT [30,31]. They conclude that in addition to canonical NE/β3AR signaling, the adenosine signaling cascade may have therapeutic benefits for both aging and obesity.

**Figure 3:**
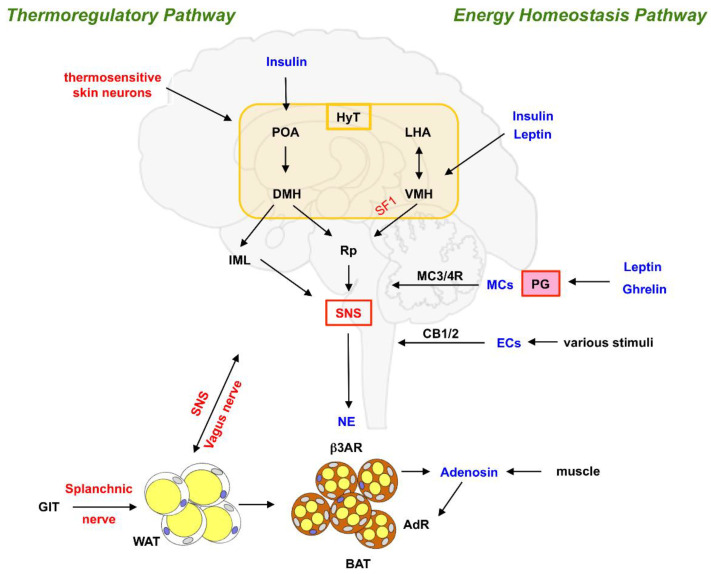
Neural circuitry controlling BAT activity. Major neuronal signaling routes are displayed in red, and molecules exerting signaling effects are shown in blue. β3AR, β3 adrenergic receptor; AdR, adenosine receptor(s); BAT, brown adipose tissue; DMH, dorsomedial hypothalamus; ECs, Endocannabinoids; GIT, gastrointestinal tract; HyT, hypothalamus; IML, intermediolateral nucleus; LHA, lateral hypothalamic area; MCs, Melanocortins; NE, norepinephrine; PG, pituitary gland; POA, preoptic area; Rp, Raphe pallidus; SNS, sympathetic nervous system; VMH, ventromedial hypothalamus; WAT, white adipose tissue.

Hypothalamic activation of this SNS-to-BAT signal is controlled by several upstream input circuits whose exact identity, mode of regulation and interdependencies are only beginning to be explored (for review, see [32]). So far, two main distinct neuronal pathways have been characterized to regulate the hypothalamic control of SNS-to-BAT signaling. The first one involves brain regions that facilitate the regulation of body temperature (termed ‘thermoregulatory pathway’), while the other one is defined by brain areas controlling the response to dietary nutrient uptake and consumption (‘energy homeostasis pathway’). Each of these circuits comprises a specific population of neurons and integrates signals from different sources (Figure 3).

As part of the **thermoregulatory pathway**, thermoreceptive neurons monitor the temperature of the skin. These neurons (which represent selective receptors for either warmth or cold) transmit thermal signals to one prominent nucleus within the hypothalamus termed the ‘preoptic area’ (POA) that serves as the central integrator of temperature signals. As demonstrated in preclinical models, cold exposure or direct hypothermic sensations within this nucleus result in POA-mediated SNS-to-BAT signaling and subsequent activation of BAT thermogenic activity [33,34,35]. The direct sensation of coldness or warmth is signaled via inhibitory GABAergic neurons within the POA, which in turn negatively regulate other neuronal subpopulations within the dorsomedial hypothalamus (DMH) [28]. Signals are further transmitted from the DMH to other CNS regions (e.g., the *Raphe pallidus* (Rp) located in the ventral tegmentum, the intermediolateral nucleus (IML) within the spinal cord), finally resulting in sympathetic activation of BAT activity. An additional level of inhibitory regulation is facilitated by neuropeptide Y (NPY) expressing neurons within the DMH, which have been discussed to inhibit BAT activity by their negative regulatory role on glutamatergic DMH neurons [28]. Importantly, these complex circuits are also under the control of adiposity signals involved in energy control since both adipose tissue-derived leptin and ß cell-derived insulin are capable of modulating the sympathetic signal, presumably by activating GABAergic neurons within the DMH [32].

A recent report by Makwana et al. complements this picture by demonstrating in a genetic mouse model that calcitonin gene-related peptide α (CGRPα)—expressing heat-sensing neurons in the skin also significantly contribute to adaptive thermogenesis and diet-induced obesity [36]. In the context of the thermoregulatory pathway, it is important to note that the contribution of BAT thermogenic activity to hyperthermia as a result of **inflammatory fever** is unresolved. Several studies used advanced model systems (e.g., Interleukin-1 beta/IL-1β dependent fever induction, RNAscope technology) to analyze the role of BAT in heat production during febrile responses. These studies identified glutamatergic neurons within the median preoptic area (MnPO) that project to the DMH to be crucial for fever-related BAT thermogenesis [37,38,39]. On the other hand, Anna Eskilsson and colleagues used Ucp-1 knock-out mice and lipopolysaccharide (LPS) administration to demonstrate that the thermogenic activity of Ucp-1 positive BAT is not involved in LPS-induced inflammatory fever [40]. Importantly, the experimental procedures to induce febrile responses and the model systems used in this study were very different and hardly comparable. It is tempting to speculate that various pro-inflammatory scenarios may result in different responses, with a divergent contribution of BAT thermogenic activity. It, therefore, remains to be elucidated how important BAT activity is for the control of body temperature during various types of fever in humans.

The hypothalamic **energy homeostasis pathway** comprises several distinct yet probably interconnected signaling systems which receive input from other extrahypothalamic brain regions (such as the nucleus of the solitary tract) to regulate food intake and energy expenditure:

The **endocannabinoid system** has recently emerged as a major contributor to the beneficial effects of bariatric surgery on metabolic improvements through its effects on thermogenesis. Endocannabinoids (EC) are endogenous fatty acid-based neurotransmitters that act via their cognate receptor proteins (endocannabinoid receptor—1/CB-1 and CB-2) in the CNS and the periphery. Responding to a plethora of stimuli (including physical exercise, food intake, glucocorticoids and general stress sensation), they mediate several vital biological functions, including regulation of energy balance, development, immune functions, mood and appetite regulation [41,42,43,44,45]. In the context of psychoactive compounds, CB-1, which is primarily expressed in the brain, is effectively activated by tetrahydrocannabinol (THC), the chemical exerting most of the psychological effects of cannabis. Under physiological conditions, CB-1 activation increases energy expenditure and mediates nutritional homeostasis via the engagement of sympathetic efferent pathways. Interestingly, CB-1 signaling has also been discussed to be involved in the regulation of epithelial barrier function in the intestine and control of gut microbiota composition [46,47]. These data argue in favor of the currently heavily discussed interaction between nutrient supply, gut microbiota composition and control of energy homeostasis via CNS-mediated neuronal circuits [48,49,50]. In a recent report, Yuanchao Ye and colleagues demonstrate that the beneficial metabolic effects of Roux-en-Y gastric bypass (RYGB) surgery in diet-induced obese mice are partly attributable to increased activity of the splanchnic nerve (a part of the SNS) by activation of CB-1 which result in visceral WAT (vWAT) browning, enhanced visceral thermogenesis and increased resting metabolic rate [51]. These observations emphasize the role of potential pharmacological modulation of SNS-associated signaling cascades.

**Melanocortins** (MCs, e.g., adrenocorticotropic hormone/ACTH, melanocyte-stimulating hormones/MSHs) represent a family of peptide hormones also involved in the regulation of food intake and energy homeostasis. Responding to the respective nutritional status and eating behavior, MC release from the pituitary gland is controlled by two important hormones which are functionally intertwined, the adipose tissue-derived **leptin** (sometimes dubbed the ‘starvation hormone’) and the ‘hunger hormone’ **ghrelin**, secreted predominantly by endocrine cells within the gastric mucosa. Secreted MCs act via binding to G-protein coupled receptor (GPCR)—type MC receptors (with MC3-R and MC4-R being mainly expressed within the brain). These receptors are both activated by the binding of α-MSH and inhibited by the intrinsic antagonist Agouti-related peptide (AgRP). Using transneural retrograde tracing by Pseudorabies virus, the group of Timothy J. Bartness has shown that SNS innervation of interscapular BAT (iBAT) originates in brain regions that significantly express MC4-R [52]. In line with this observation, the injection of an MC4-R agonist directly into the brain is capable to activate iBAT thermogenesis [45,46]. Focusing on the link between CNS and white adipocytes, Jenna Holland and colleagues demonstrated that Mcr-4^-/-^ mice with reduced brain MC signaling experience gain in WAT fat mass due to increased lipogenesis and proliferation of WAT cells. These effects were conveyed by efferent innervation of the **vagus nerve**, as shown by subdiaphragmatic vagotomy [53]. The vagus nerve represents the longest and probably most important nerve of the parasympathetic system and controls various vital organ functions, such as heart activity and digestion. The authors could demonstrate that vagal signals contribute to obesity caused either by Mcr-4 deficiency or high-fat feeding. Intriguingly, as demonstrated by pair-feeding of wildtype and Mcr-4 knock-out animals, the vagus-mediated gain in fat mass was independent of caloric intake. These data emphasize the role of the vagal signals for energy homeostasis and may demonstrate that brain-melanocortin signaling controls the fat mass of the organism at least by two mechanisms, indirectly by controlling energy balance and directly by SNS-mediated lipid mobilization in BAT.

It is important to note that most of the above-mentioned experiments concerning MC signaling were performed in hibernating animals whose major BAT depot is located in the interscapular region. As evidence for the functionality of iBAT depots in human adults is still limited, the scope to which these mechanistic insights of BAT activation via MCs from rodent models could be used for the treatment of metabolic disease in humans is still unclear (for critical review see [54]). On the other hand, since the MC system represents one of the pivotal downstream signaling axes activated by leptin, it is tempting to speculate that involvement of leptin gene variants or impaired leptin receptor signaling in early onset obesity [55] may at least partially be promoted by impaired crosstalk between leptin and MC signaling cascades. These preclinical data could underscore the potential role of leptin-controlled MC signaling as a contributor to SNS-mediated BAT activity, suggesting MC receptor signaling also as a potentially interesting drug target for the treatment of metabolic disorders (for review see [56]).

Although the POA and dorsomedial hypothalamus (DMH) were originally considered to be the major hypothalamic nuclei in the thermoregulatory network [27], recent molecular studies further point toward the lateral hypothalamic area (LHA) and the ventromedial hypothalamus (VMH) to also be involved in BAT regulation [57]. The VMH harbors neurons that are characterized by highly specific expression of the developmentally essential transcription factor SF1 (steroidogenic factor 1). Genetic ablation of SF1 neurons (another essential component of the hypothalamic energy homeostasis pathway) in sophisticated model systems demonstrates that this VMH-residing neuronal population controls energy expenditure, BAT architecture and thermogenic activity [58,59]. While retrograde viral tracing was not suitable to show direct VMH-to-BAT neuronal linkage [52], it is currently speculated that the signal from VMH may be relayed by hindbrain structures (such as the *Raphe pallidus*) or even regions in the brain stem (e.g., the nucleus of the solitary tract). Mechanistically, VMH-to-BAT signaling via SF1 neurons is dependent on AMPK and mTOR signaling pathways and is effectively activated by both leptin and insulin (for review: [28]). This also demonstrates that, for the purpose of BAT activation, both main pathways (thermoregulation and energy homeostasis) closely interact via hypothalamic integration.

As sketched above, BAT thermogenesis is not exclusively controlled by hypothalamic signaling via the SNS alone, but a variety of other neuronal populations in other regions of the CNS appear to contribute to the hormonal regulation of BAT-mediated thermogenesis. These include hindbrain neurons [60], GABAergic neurons in the brainstem [61] and parapyramidal neurons within the medullary raphe, which are negatively regulated by midbrain GABAergic cells within the substantia nigra [62]. The complex interplay between these neuronal subpopulations and their intrinsic activating and/or inhibitory crosstalk warrants further investigation to fully understand (and potentially harness) neuronal control of thermogenic activity in the context of obesity.

While the neuronal control of BAT activity by the CNS is well established in preclinical models, recent data may point to an unexpected direct neuronal link between the two major adipose tissue types: Using a Siberian hamster model Garretson and colleagues analyzed BAT innervation by neurons residing within WAT depots. They tested the hypothesis that lipolysis in WAT activated neuronal activity of WAT afferent neurons and demonstrated that lipolysis-dependent fFA production triggers neuronal activity in WAT, which is capable of activating BAT thermogenesis [63]. This mechanism may have important implications, especially under conditions that favor high levels of lipolysis, such as cold exposure, thus warranting further investigation as to its level of translation to the human disease state.

### 2.2. Hormonal Regulation of BAT Activation

In addition to the CNS, a number of non-neuronal endocrine signaling systems have emerged as important players in the control of BAT thermogenic activity. Interestingly, they derive primarily from the gastrointestinal (GI) system. The involvement of the GI tract and its signaling to the brain in multiple aspects of energy homeostasis and metabolic control has clearly been demonstrated by the impressive clinical success of bariatric surgical procedures. In fact, nothing currently available is nearly as effective in yielding sustained weight management and improvement of cardiometabolic complications. Adaptive mechanisms of the intestine involving increased numbers of endocrine cells have raised enormous interest in the possibly therapeutic role of postoperatively altered gut signals that act in the brain and other organs to modulate energy balance and metabolic control. Only within the last two decades have the molecular mechanisms of appetite control advanced to a point where drug discovery can be rationally pursued with suitable tolerability and safety [64].

In fact, recent clinical trials with advanced therapeutic candidates, including the gut-derived glucagon-like peptide 1 (GLP1)-, the glucose-dependent insulinotropic polypeptide (GIP)- and the pancreatic glucagon (GCG)-receptor signaling agonists arouse huge clinical expectations as to successful weight management based on carefully designed drug combinations. The current weight loss drugs in clinical development signal via specific cognate receptors, which all belong to the G-protein coupled receptor (GPCR) family and thus represent attractive pharmacological targets. Importantly, while the downstream signaling mechanisms of the three hormones are very similar, the expression profile of the cognate receptors is highly specific and thereby conveys their tissue-specific effects.

The peptide hormone Glucagon/GCG is produced by alpha cells of the pancreas and represents the main catabolic hormone of the human body. Together, insulin and GCG control glucose levels in a counter-regulatory manner. The Glucagon receptor (GCGR) is expressed dominantly in the liver (and to a minor extent in the kidneys) but not in the CNS. After engagement by its ligand, GCGR mediates hepatic glucose production via hydrolysis of glycogen and gluconeogenesis. GCG has also been shown to increase energy expenditure and influence BAT function under cold exposure conditions, i.e., during adaptive thermogenesis [65,66]. As a potential mechanism for this effect, GCG-mediated secretion of Fibroblast Growth Factor 12 and bile acids from the liver and downstream engagement of BAT have been discussed [65].

Based on their shared function in orchestrating post-prandial glucose- and lipid metabolism, GLP-1 and its sister hormone, GIP, are collectively referred to as **incretins** [67,68,69]. GLP1 is a 30-amino acid peptide produced in the intestinal epithelial endocrine L-cells by differential processing of pro-glucagon, the same precursor protein that gives rise to GCG. GLP-1 mainly acts by stimulating insulin and inhibiting glucagon secretion by the pancreas. In contrast to GCGR, the GLP-1 receptor (GLP-1R) is prominently expressed not only in pancreatic cells but also in the hypothalamus, where its signaling controls appetite and food intake. The hypothalamic expression of GLP-1R significantly contributes to the central role of GLP-1 in regulating BAT thermogenesis in response to the nutrient status but also by browning effects in WAT [66,70,71]. GIP is produced by enteroendocrine K-cells and is released as a response to increased nutrient status. While it is a weak inhibitor of gastric acid secretion, its main function is to exert incretin activity in a glucose-dependent manner along with its sister hormone GLP-1, i.e., to augment insulin secretion and drive utilization and disposal of post-prandial blood sugar excursions. GIPR is highly expressed in several tissues, including the stomach, pancreas, heart muscle, neuronal tissues and fat tissue. In the CNS, GIPR expression can be found in almost all brain areas [72]. Importantly, a contribution of GIP signaling to BAT thermogenesis is expected but has to date not been demonstrated experimentally.

These three molecules, GLP-1, GIP and GCG, are currently taking center stage in promising drug development strategies focusing on obesity and diabetes. The current status of this fascinating quest for novel therapies is further described in chapter 3.

Another major breakthrough in understanding nutrient handling was made by recent findings of Martin Klingenspor’s group, which demonstrated that food-dependent release of the gut hormone **secretin** results in the activation of secretin receptors on BAT adipocytes [73]. Stimulation of the receptor signaling pathway induces BAT lipolysis and post-prandial thermogenesis, finally resulting in increased cellular glucose uptake and sensation of satiety induced by secretin-dependent BAT-activation. This study thereby established BAT as a post-prandial satiety-mediating organ triggered by food-dependent signals such as secretins. It will be interesting to investigate which other gut-derived signals are able to trigger this inter-organ crosstalk and how these pathways are controlled or impaired under physiological and pathological conditions, respectively.

Given the tight interaction between nutrient uptake, energy homeostasis and hormonal control of metabolic traits, it is not surprising that hormonal signals from the gastrointestinal tract and the thyroid gland (thyroid hormones/THs, i.e., thyroxine/T4 and its bioactive product triiodothyronine/T3) are pivotally involved in BAT activation. The TH receptor expression is high in BAT, and activation of these receptors results in the upregulation of UCP1 and amplification of NE–mediated BAT thermogenesis (review: [74]). The importance of human disorders is further underscored by the findings that chronic exposure to TH results in enhanced BAT mass and activity, as well as improved metabolic control of the response to glucose [75].

### 2.3. Additional Mechanisms Regulating BAT Activity

Another currently fervently discussed concept in line with the gut-adipose tissue crosstalk is the capability of the intestinal microbiome (iMB, i.e., the entirety of several trillions of microorganisms, including bacteria, archaea, fungi and viruses residing within our intestines) to modulate adipose tissue activity effectively. It is well accepted that the iMB (sometimes considered as an additional metabolic ‘organ’) represents an important modulator of host metabolism and thus contributes to the modulation of the health status of its host. By taking up and converting nutrient components by anaerobic fermentation, the iMB produces and provides a plethora of metabolites to the portal vein system and systemic circulation. These metabolites include short-chain fatty acids (such as butyrate and acetate), branched-chain amino acids and secondary bile acids (such as glycine- or taurine- conjugates). By activating GPCR-mediated signaling cascades on host cells, microbial metabolites can affect their host’s nutrient and energy balance. In particular, rodent models have provided evidence that alterations in the gut microbiota composition (rather than the presence or absence of one particular bacterial taxon) affect the thermogenic program in BAT and modulate browning effects in WAT, presumably via altered metabolite signaling and/or by the involvement of the endocannabinoid system (reviewed in [76]). Moreover, there is growing evidence showing that iMB composition also affects metabolic dysfunction linked to comorbidities such as cardiovascular diseases [77]. While the concept of a direct association between iMB structure and BAT activity in humans is still being debated [78], the accessibility to targeted, non-invasive modulation (e.g., by fecal microbiota transfer/FMT or spiking-in of selected bacterial communities with beneficial effects on host metabolism) still renders the gut microbiome an attractive target for therapeutic interventions [79].

Finally, novel findings suggest that BAT exhibits circadian rhythms in its thermogenic activity and is entrained into the environment via external cues (called “zeitgebers”) such as light exposure and timed food intake. While several routes of control may exist [72,73,74], it was recently demonstrated by Orozco-Solis and colleagues [80] that the circadian clock region within the VMH integrates dark-light cycle oscillations and food intake behavior to control BAT thermogenesis via the SNS. Using neuronal subtype-specific knock out of the core clock gene *Bmal1* in mice, they uncovered the underlying mechanism, demonstrating that the observed oscillations in BAT activity over 24 h day–night cycles are mediated by clock genes such as *RevErb-alpha* which (controlled by master circadian rhythm genes such as *Clock* and *Bmal1*) acts as a transcriptional inhibitor of *Ucp1* [81], thus leading to oscillatory *Ucp1* abundance and activity.

Taken together, multiple pathways converge to control the activity of BAT cells, thus integrating various extrinsic and intrinsic signaling cues. However, does this communication network also work in the opposite direction?

### 2.4. Feedback from BAT to Brain

While the scientific focus layed a long time on the outgoing signals from CNS to functional BAT control, it is clearly less well understood how fat tissue returns activation signals to the CNS in order to orchestrate energy homeostasis. Sophisticated transneuronal tracing tools involving specific strains of herpes simplex virus-1 (HSV-1) that travel anterogradely along the outflow of sensory nerve signals have been utilized to study potential feedback circuits between fat tissues and the brain [60,82,83,84]. As shown by Song and colleagues [82] and further described by Bartness et al. [60], one general mechanism for vital WAT-to-brain feedback is mediated by sensory neurons that innervate WAT and afferent signaling to dorsal route ganglia (DRG). Moreover, recent studies support the existence of SNS-mediated sensory feedback between BAT and the CNS, pointing to areas in the brain stem and the forebrain as target regions of these signals [60]. Vitaly Ryu and colleagues used a combination of retrograde pseudorabies virus and anterograde HSV-1 tracing to demonstrate that BAT is innervated by sensory neurons, which potentially signal changes in temperature (caused by BAT thermogenic activity) via DRG to several brain regions involved in energy homeostasis, e.g., the *raphe pallidus* and the *hypothalamic paraventricular nucleus* [83]. Cheryl and Bartness also used anterograde transneuronal viral tract tracing in Siberian hamsters to analyze the role of sensory circuits from BAT to the CNS for body temperature control. Depletion of sensory neurons originating in iBAT by injection of the neurotoxin capsaicin in combination with cold exposure treatment convincingly demonstrated that calcitonin gene-related peptide (CGRP)/substance P—positive sensory neurons constitute components of a central sensory circuit from BAT to the CNS involved in thermogenesis [84]. Taken together, the fat tissue is not only receiving input from the CNS but, importantly, is also providing sensory feedback to orchestrate storage or mobilization of lipids, thus conveying energy homeostasis. It is important to note that these feedback mechanisms, while potentially forming the basis for future therapeutic strategies, are far less understood than the signals going out from the brain to BAT, thus urgently calling for further investigations.

## 3. BAT—A Promising Target for Management of Obesity-Related Diseases?

Over the last 50 years, we have faced a world-wide and further proceeding epidemic of obesity, which is essentially driven by a chronic energetic imbalance where energy intake chronically exceeds energy expenditure. All efforts to prevent a further rise in prevalence, together with currently available pharmacological anti-obesity strategies, have proven ineffective in solving the pandemic development. All the more has the control of excess ectopic fat depots become one of the greatest healthcare challenges of our time, and the demand for novel weight-centric strategies as a primary treatment goal for obesity-associated cardiometabolic diseases is high. The rediscovery of functionally active BAT in adults almost 15 years ago triggered a resurgence in BAT research combined with the hope to exploit physiological thermogenesis to achieve the necessary net energy balance required for sustained weight management (see summary in Table 1).

While the past decades have witnessed great advances in the mechanistic understanding of cellular thermogenesis and revealed an important role of thermogenic dissipation of excess calories in modulating systemic energy homeostasis and recovery of metabolic health in *preclinical disease models*, the clinical translation of these findings as a possibly future complementary therapeutic approach to *human obesity and cardiometabolic diseases* is still its infancy (for detailed review, see [85]). Overall in humans, BAT contribution to thermogenesis has been reported to range from 27–123 kcal per day at room temperature to 46–211 kcal per day during mild cold exposure [86], whereas fully activated BAT could dissipate energy equivalent to approximately 4 kg of WAT over one year [15]. Interestingly, a recent large epidemiological study indicates that BAT presence in humans inversely correlates with lower blood glucose and triglyceride levels, higher HDL levels, and improved cardiometabolic health [18]. As to its overall energetic function, BAT activity has been reported to correlate with leanness [3] and fat liver content [13]. Moreover, subjects with more active BAT exhibit improved metabolic profiles [14] as well as reduced arterial inflammation and calcification [17]. Interestingly, the reported beneficial metabolic effects of BAT presence/activity in humans may not be necessarily related to adiposity reduction. Recent data indicate that pharmacological, chronic BAT activation increases insulin sensitivity and glucose effectiveness as well as high-density lipoprotein and adiponectin levels in the blood, even without significant changes in body weight or body composition [87]. Thus, while studies in humans suggest several positive effects of BAT activation on energy homeostasis and metabolic outcome parameters, its fundamental physiological role and clinical significance as a putative novel therapeutic target in the context of obesity and related cardiometabolic diseases is still under investigation. Today more than 100 clinical have been conducted focusing on human BAT as a therapeutic target for obesity and related cardiometabolic diseases. The intended approaches are either targeting directly the thermogenic activity or the recruitment of BAT mass in patients with obesity [6,7,88] combined with the hope that pharmacological regulation of thermogenesis will not only support a reduction in visceral obesity but also improve or even prevent subsequent cardiometabolic dysfunctions.

The early and conceptual appeal to pharmacologically activate BAT metabolic activity via sympathomimetics and enforced induction of β3AR signaling was mainly hampered by adverse effects on the cardiovascular system (as exemplified by the dramatic effects of adrenergic receptor agonists like ephedrine [89] and mirabegron [90]) and dissimilarities in receptor structure and engagement [91,92]. In particular, the potential positive effects of sympathetic activation on energy expenditure and fat mass reduction by β3AR agonists (or supraphysiological thyroid hormone supplementation) are voided by deleterious effects on the cardiovascular system, namely elevation in heart rate and blood pressure. This is even more relevant in the cohort of obese patients with substantially increased cardiovascular risk and has led to the sub sequential failure of several weight loss drugs in the past.

Up to date, controlled cold exposure is the best-studied therapeutic approach for BAT activation in human subjects (see expert review: [93]). However, the studies conducted so far were only short-term by design, and potential application for a wide set of patients might be unfeasible. Moreover, a compensatory increase in caloric intake has to be considered (such as seen in cold-induced thermogeneration models in mice [94] and male human subjects [95]). More research is needed to better understand the implications of controlled cold exposure as a means to increase resting energy expenditure and reduce fat mass.

**Table 1:** BAT-based therapeutic strategies under investigation for treatment of obesity and/or cardiometabolic diseases. See main text for details.

Therapeutic Strategy	Status (2022)	References
Enforced adrenergic signaling via β3AR agonists	hampered by deleterious effects on cardiovascular system	[89,90,91,92,93]
BAT activation/WAT depletion by thermogenesis-promoting diet, supplements or physical exercise	Pilot studies, promising, safe, feasibility has to be tested	[85]
BAT activation/WAT depletion via controlled cold exposure	promising, safe, but feasibility has to be tested	[93]
Targeting Melanocortin receptors (downstream of leptin)	pleiotropic function of MCs questions applicability	[56]
Mimicking incretins (GLP-1, GIP mimetics)	promising strategy, first results from clinical studies are encouraging	[70]
Designing Triple agonists (mimicking GLP-1, GIP, GCG in a single molecule)	clinical trials successful, awaiting market entry	[96,97,98,99,100,101,102]
BAT transplantation	Pilot studies, experimental	[85]
Mediating differentiation of pre-adipocytes/progenitor cells/stem cells	Pilot studies, experimental	[85]
Targeted modulation of the gut microbiome	Pilot studies, experimental	[85]
Altering gut metabolite and/or bile acid profiles	Pilot studies, experimental	[85]
Precise modulation of gut-to-brain/brain-to-BAT signaling	Pilot studies, experimental	[85]
Promotion of WAT beiging by batokines, metabolites, pharmacological agents and/or miRNAs	Pilot studies, experimental	[85]
Activation of thermogenesis in WAT and/or muscle	Pilot studies, experimental	[85]

One current strategy focusses on targeting Melanocortin receptors as potential drug targets in the context of obesity and eating disorders [56]. This is motivated by the role of MC signaling as an effective downstream effector of leptin, but also by the multitude of biological processes regulated by MCs, encompassing energy homeostasis but also inflammatory signaling, regulation of blood pressure, pain sensation and behavioral traits.

The emerging role of incretin hormones (GLP-1, GIP) and the insulin-counteracting Glucagon/GCG on metabolic function has sparked huge interest in targeting the associated GPCR-mediated pathways for therapeutic purposes. In the context of obesity, GLP-1 and its receptor GLP-1R have prominently entered center stage in the pharmacological treatment of T2D and obesity. Sequence-optimized GLP-1 agonists (such as Semaglutide and Liraglutide) have been shown to induce weight loss and improvement of glucose homeostasis via complementary signaling effects, like modulation of hunger and satiety sensation, delaying gastric emptying, facilitating insulin secretion and decrease of hepatic gluconeogenesis. However, BAT activation and thermogenesis also take part in the orchestration of GLP-1-induced weight loss. For example, it was demonstrated in mice that the central application of Liraglutide induced BAT thermogenesis and WAT browning [70]. Moreover, in obese T2D patients, Liraglutide and Exenatide increased resting energy expenditure independently from physical activity [70].

Intriguingly, recent developments in drug development focus on the use of molecules that engage not only one but multiple relevant pathways at the same time to harness the complementary metabolic effects of specific signaling pathways via this complementary approach. These efforts aimed to design, optimize and finally apply single molecules that activate GLP-1R in parallel to receptors for GIP and/or GCG to optimize effects on nutrient uptake, lipolysis and energy expenditure. Chimeric peptides resembling a molecular mosaic of two or three of these ligands (dubbed ‘dual’ (GG) and ‘triple’ (GGG) agonists), which are chemically modified for improved stability and uptake, have successfully passed clinical trials and/or are awaiting approval for clinical use and market entry [96]. By adding the anorectic, lipolytic and insulinotropic properties of these agents in a single molecule, effects on weight loss and glucose metabolism are maximized without the disadvantages of polypharmacy at the cost of tolerability. In preclinical models, it had been shown before that dual GLP-1/GCG as well as dual GLP-1/GIP agonists improve metabolic parameters and are capable of fighting obesity [97,98]. Moreover, a rationally constructed tri-agonist that mimics the activity of GLP-1, GIP and GCG in one single poly-receptor agonist is able to dramatically improve the outcome of both obesity and diabetes [99]. Proving the clinical applicability of this concept, the dual agonist Tirzepatide (TZP), a GIP/GLP-1 analog that shows higher affinity to GIPR than to GLP-1R, demonstrated safety and efficacy by improving glycemic control and weight management in a clinical trial program for T2D [100]. Moreover, TZP was superior to the mono-agonistic drug Semaglutid with respect to glycemic control [101] and successfully demonstrated profound effects on body weight reduction over a 72-week trial in obese patients without diabetes [102]. This series of successful clinical studies resulted in the approval of TZP in combination with diet and exercise for the improvement of blood sugar control in adults with T2D by the U.S. Food and Drug Administration (FDA) in May 2022. These promising data suggest that it may not take too long until the first GLP-1/GIP/GCG triple receptor agonist passes registration and enters the market for the treatment of obesity and diabetes.

It is important to note that the three receptor types display a very distinct expression profile which may result in organ-specific effects of the chimeric drugs, depending on the exact combinatorial structure. This phenomenon is part of the drug design strategy but may also lead to unexpected cross-reactions (For details on this fascinating topic, we refer the reader to the expert review by Capozzi et al. [96]). Nevertheless, it is anticipated that the current clinical trials aiming at the approval of dual and triple agonists for metabolic disorders will demonstrate the usability of this approach in the very near future. It will be interesting and important to see how these next-generation anti-obesity drugs will deal with safety and long-term efficacy concerns. The biggest challenge for upcoming pharmacological approaches is to combat the organism’s homeostatic drives to return the weight back to higher levels over time (metabolic adaption). Despite a seemingly simple equation (“energy in less than energy out”) it remains difficult to achieve long-term weight loss as the body tends to adapt or resist by modulating energy expenditure, hormonal and psychological drive.

In this context, it is also noteworthy that the combined effect of these designer drugs on several additional pathologically relevant pathways (e.g., oxidative stress, inflammation and cellular proteostasis) may, in the future, also be relevant not only for targeting BAT as a therapeutic option against obesity but also for fighting other diseases types, e.g., neurodegenerative disorders like Alzheimer Disease [103,104].

The engagement of BAT as a therapeutic target in metabolic diseases implies several advantages but also some disadvantages (for an in-depth review, see [88]): On the up side, there lies a great chance in utilizing BAT’s broad metabolic adaptation potential for the treatment of metabolic disorders, especially in approaches that aim at increasing activity and/or mass of brown adipocytes (e.g., via browning or beiging of WAT). In line with this concept, additional BAT functions such as glucose usage and lipolysis are also beneficial for metabolic improvement [54,105,106]. On the other hand, β3-R agonists failed in human trials due to receptor sequence dissimilarities and adverse effects such as tachycardia caused by the β3-R expression on cardiac myocytes [90], as outlined above. Finally, there is a certain risk of a compensatory increase in energy intake.

It follows from these observations that it may be useful to conceptualize the pharmacological treatment of obesity diseases as one would approach other chronic conditions, such as essential hypertension or T2D. Just as hypertension is often treated with combinations of sympatholytics, ion channel blockers, and enzyme inhibitors, the pharmacological approach to obesity will likely require a combination of drugs that suppress appetite, impair absorption and increase energy expenditure in parallel (or at least in an orchestrated manner). While a BAT-centric approach may be one facet in this composite endeavor, the above-mentioned pitfalls render this strategy challenging, at last, unless pharmacological intervention succeeds in designing non-adrenergic signaling control of BAT thermogenesis. Interestingly, there is a number of alternative BAT-centered approaches that could harness the knowledge of underlying BAT-regulating mechanisms for the design of successful therapy. These include BAT transplantation, usage of progenitor or stem cells that give rise to brown adipocytes (or precursor cells that can be further differentiated), modulation of the gut-brain signaling axis either by targeted shifts in the gut microbiome or by tweaking of metabolite or bile acid profiles, or precise modulation of brain-to-BAT signaling (or its feedback signaling route) by tailored agonists of respective signaling pathways. Along this line, a major aim of current and future therapeutic approaches would thus be to harness known neural signal routes in order to fine-tune BAT activity in obese patients. Consequently, drugs that fine-tune CNS to BAT neuronal signaling or feedback signaling will open avenues for therapy or even prevention of obesity and its comorbidities. Intriguingly, there is another fascinating perspective: The recent realization that cold-induced thermogenesis is not exclusively restricted to BAT but is also happening in beige adipose tissue and in muscle (reviewed in [6]) may provide another entry point to therapeutic strategies in the future. It is still important to note that the current knowledgebase is insufficient to provide a rationale for respective approaches and will require more in-depth studies on this fascinating option.

In conclusion, as non-surgical therapies for obesity are urgently needed, harnessing the growing knowledge about brain-to-BAT-to-brain signaling in combination with other controlling instances may provide promising future therapies for metabolic diseases. One major task will be the identification of feedback mechanisms, e.g., how body weight and the energy storage state are monitored within the body and integrated into the CNS to orchestrate energy expenditure and homeostasis. Further research in this area, leading to a detailed understanding of this ‘Baristat’ and its regulatory mechanisms, will help us to identify promising targets and open novel avenues for therapies against the imminent obesity pandemic.

## Abbreviations

18FDG: 18F-fluoro-2-deoxy-d-glucose; AAC, ADP/ATP carrier; ACTH, adrenocorticotropic hormone; AgRP, Agouti-related peptide; ATGL, adipose triglyceride lipase; β3AR, β3 adrenergic receptor; BAs, bile acids, BAT, brown adipose tissue; BCAA, branch-chained amino acids; BMI, body mass index; CGRP, calcitonin gene-related peptide; CNS, central nervous system; CT, computed tomography; DMH, dorsomedial hypothalamus; DRG, dorsal route ganglia; EC, Endocannabinoid(s); ER, endoplasmatic reticulum; FDA, U.S. Food and Drug Administration; fFAs, free fatty acids; FGF, fibroblast growth factor; GCG, Glucagon; GCGR, Glucagon receptor; GIP, gastric inhibitory peptide; GIT, gastrointestinal tract; GLP-1 Glucagon-like protein-1; GLUT, Glucose transporter; GPCR, G-protein coupled receptor; HSL, hormone-sensitive lipase; HSV-1, herpes simplex virus-1; HyT, hypothalamus; iBAT, interscapular BAT; IGF-1, insulin-like growth factor 1; IL-6, interleukin 6; iMB, intestinal microbiome; IML, intermediolateral nucleus; LPS, lipopolysaccharide; MC, Melanocortin; MnOP, median preoptic area; MSH, melanocyte-stimulating hormones; NE, norepinephrine, PET, positron emission tomography; PG, pituitary gland; PKA, protein kinase A; POA, preoptic area; Rp, Raphe pallidus; RBP-4, retinol binding protein 4; RYGB, Rous-en-Y bypass surgery; SCFA, short-chained fatty acids; secBA, seconday bile acids; SNS, sympathetic nervous system; T2D, type 2 diabetes; TG, triglycerides; TGF-beta, transforming growth factor beta; TH, thyroid hormone; THC, tetrahydrocannabinol; TZP, Tirzepatide; VMH, ventromedial hypothalamus; WAT, white adipose tissue; WHO, World Health Organization.
